# Evaluating the lifetime performance index of omega distribution based on progressive type-II censored samples

**DOI:** 10.1038/s41598-024-55511-w

**Published:** 2024-03-08

**Authors:** N. M. Kilany, Lobna H. El-Refai

**Affiliations:** Department of Mathematics and Computer Science, Faculty of Science, Menoufia University, Shebin El-Kom, Egypt

**Keywords:** Process capability indices, Omega distribution, Progressive type-II censored sample, Lifetime performance Index, Markov Chain Monte Carlo, Bayes estimation, Mathematics and computing, Statistics

## Abstract

Besides achieving high quality products, statistical techniques are applied in many fields associated with health such as medicine, biology and etc. Adhering to the quality performance of an item to the desired level is a very important issue in various fields. Process capability indices play a vital role in evaluating the performance of an item. In this paper, the larger-the-better process capability index for the three-parameter Omega model based on progressive type-II censoring sample is calculated. On the basis of progressive type-II censoring the statistical inference about process capability index is carried out through the maximum likelihood. Also, the confidence interval is proposed and the hypothesis test for estimating the lifetime performance of products. Gibbs within Metropolis–Hasting samplers procedure is used for performing Markov Chain Monte Carlo (MCMC) technique to achieve Bayes estimation for unknown parameters. Simulation study is calculated to show that Omega distribution's performance is more effective. At the end of this paper, there are two real-life applications, one of them is about high-performance liquid chromatography (HPLC) data of blood samples from organ transplant recipients. The other application is about real-life data of ball bearing data. These applications are used to illustrate the importance of Omega distribution in lifetime data analysis.

## Introduction

To guarantee the precision, dependability, and utility of data, quality control is an essential procedure. In order to detect and reduce biases, errors, and inconsistencies in data collection, processing, and interpretation, a variety of methods and instruments are used. To achieve quality control, a variety of methods and instruments are available, including process control charts and capability analysis. The three key process variables that control charts track are mean, range, and standard deviation. Any significant deviations from normal patterns indicate potential issues requiring investigation and corrective action. There are many articles related to this point. Conditional mean- and median-based cumulative sum control charts were calculated for Weibull data by Raza et al.^1^. Ali et al.^2^ showed the effect of estimation error for the risk-adjusted Charts,(see also Refs.^3–5^ and Ref.^6^). Rather than assessing the process's overall quality, a control chart tracks the consistency of the product quality generated inside a certain manufacturing process. Although control charts are widely used in the industry, real-time process capability estimation and quality monitoring are not possible with them.

Because products differ in terms of specifications and units, managers must first determine target values and associated tolerances in order to assess process capabilities. Process capability indices are essential instruments for evaluating a process's capacity to produce a good that complies with requirements. The industry's most popular method for evaluating process quality is these indicators. This paper dealt with one of the process capability indices to test its conformity with quality specifications, which is the lifetime performance index.

Many services under certain specifications (desired level) are desired from customers. Modern institutes assess the quality performance of items to provide special needs to their customers. Statistical methods are used to control and promote the quality performance of items. Therefore, process capability indices (PCIs) are employed to detect whether the quality performance of an item reaches the desired level. Montgomery^7^ and Kane^8^ illustrate various PCIs in literature. ${C}_{L}$ is utilized by assessing the performance of electronic components lifetime, where $L$ is the lower specification limit. The performance of electronic components lifetimes is determined by employing the larger-the-better process capability index ${C}_{L}$.

Recently, the lifetime performance index ${C}_{L}$ is used in many research for various fields.  Using data from High-Performance Liquid Chromatography (HPLC) of blood samples from organ transplant recipients, Rady et al. ^9^ investigated the statistical inference of the lifetime performance index for the Topp Leone Alpha power exponential distribution based on first failure progressive censoring schemes. El-sagheer et al.^10^ provide an assessment for the lifetime performance index of power hazard function distribution. Ahmadi and Doostparast^11^ evaluate ${C}_{L}$ for Pareto distribution with a new Bayesian approach. Inference of ${C}_{L}$ for Power Rayleigh distribution is provided by Mahmoud et al.^12^ on progressive first failure censored sample. Hassan and Assar^13^ evaluate the lifetime performance index of Burr type III distribution under progressive type II censoring. Hassanein^14^ measured ${C}_{L}$ for Lindley distribution on progressive first failure censoring data. Assessing the lifetime performance index of Weighted Lomax distribution under progressive type-II censoring scheme for bladder cancer is presented by Ramadan^15^. Wu and Hsieh^16^ assess ${C}_{L}$ with Gompertz distribution on Progressive type-I interval censored sample. Wu et al.^17^ calculate the lifetime performance index for Weibull products. Inference of ${C}_{L}$ of Gamma distribution by point and interval estimation is constructed by Shaabani and Jafari^18^. A credible interval for ${C}_{L}$ of Rayleigh products based on Bayesian estimation is calculated by Lee et al.^19^. Also, see Laumen and Cramer^20^. Statistical inference for the lifetime performance index of products with Pareto distribution under general progressive type-II censored sample is measured by Zhang and Gui^21^. Ahmad et al.^22^ investigate the lifetime performance index under Ishita distribution based on progressive type II censored data with applications. The lifetime performance index for Stacy distribution applied to medical and engineering data by Elhaddad et al.^23^.

Moreover, institutes strive to save costs and time of testing. Censored data can be used for solving these problems. In survival analysis, many censoring schemes are proposed. Progressive type-II censoring is the most fundamental type in survival analysis. Progressive type-II censored sample can be described as follows,

- Assume that $n$ randomly specified items are set at time zero on a test and at the time of the $m-th$ failure, the test finished.
- ${R}_{i}$ of the surviving items are removed randomly from the test, when the $i-th$ product fails $(i=1,\dots . ,m-1).$
- At the end of the test, all ${R}_{m}$(items which are still surviving) are ejected from the test when the $m-th$ failure occurs.

**Figure 1:**
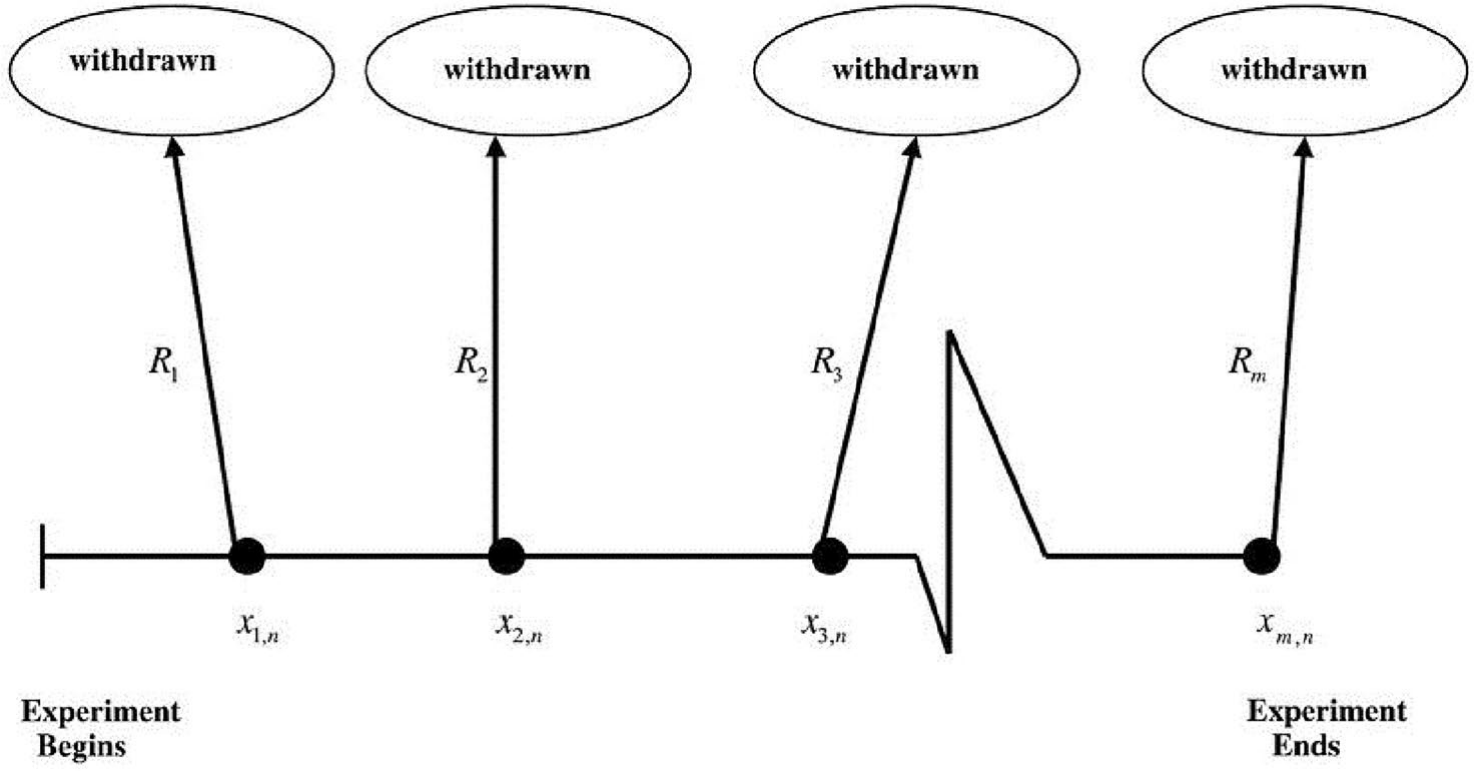
The framework of a progressive type-II censoring scheme.

Note that, $m$ and $R= ({R}_{1}, \dots \dots , {R}_{m})$ are pre-defined and $\sum_{i=1}^{m}{R}_{i}=n-m$. Also, if ${R}_{i}=0$ for $\left(i=1,\dots . ,m-1\right)$ and thus ${R}_{m}=n-m,$ the progressive Type-II censoring scheme is abbreviated to the Type-II censoring scheme and if ${R}_{i}=0$ for $i=1,\dots . ,m-1$, this censoring scheme is simplified to the complete sample; see Balakrishnan^24^. Figure 1 shows the mechanism of progressive type-II censored data.

In this paper, the items lifetime distribution may not obey the normal distribution. The omega distribution is a relatively new probability distribution with three parameters $(\alpha , \beta , and \gamma )$ that can be used to model various hazard function shapes, including the bathtub shape. We compare the omega distribution's performance with other models to show its effectiveness and potential advantages in modeling bathtub-shaped hazard function. Omega distribution introduced by Dombi et al.^25^ is used to measure ${C}_{L}$ to detect if the quality of the item meets the desired level or not. Assume that $X$ be random variable which satisfy omega model with three parameters $\alpha ,\beta ,\gamma >0$. The probability density function (pdf) and the cumulative distribution function (cdf) of the Omega distribution are constructed as follows,

1 $$f\left(x\right)=\frac{\alpha \beta {x}^{\beta -1}{\gamma }^{2\beta }}{{\gamma }^{2\beta }-{x}^{2\beta }}{(\frac{{\gamma }^{\beta }+{x}^{\beta }}{{\gamma }^{\beta }-{x}^{\beta }})}^{\frac{-\alpha {\gamma }^{\beta }}{2}}, \,\,0<x<\gamma , \,\,\alpha >0,\,\, \beta >0,\,\, \gamma >0$$

2 $$F\left(x\right)=1-{\left(\frac{{\gamma }^{\beta }+{x}^{\beta }}{{\gamma }^{\beta }-{x}^{\beta }}\right)}^{\frac{-\alpha {\gamma }^{\beta }}{2}}, \,\,0<x<\gamma , \,\,\alpha >0,\,\, \beta >0,\,\, \gamma >0$$

The rest of this paper, Sect. "The lifetime performance index of Omega distribution" represented the lifetime performance index of Omega distribution. The conforming rate is calculated for the items in Sect. "The conforming rate". Section "Maximum likelihood estimator of lifetime performance index" introduced the maximum likelihood estimation of the ${C}_{L}$. Section "Bayesian estimator of the lifetime performance index" introduce Bayesian estimation for unknown parameters and ${C}_{L}$. Section "The testing procedure for the lifetime performance index" involved testing procedure of the ${C}_{L}$. Simulation study is done in Sect. "Simulation study". Two real data sets HPLC data and Ball Bearing data are given in Sect. "Applications".

## The lifetime performance index of Omega distribution

In order to satisfy customers' demands, the lifetime of products should exceed the lower specification limit known as $L,$ since the products lifetime exhibit the larger-the-better quality characteristic. Suppose that $X$ the product lifetime and $X$ constitute by the Omega model with the pdf and cdf shown in Eqs. (1) and (2). Then, ${C}_{L}$ is defined on the next equation,

3 $${C}_{L}=\frac{\mu -L}{\sigma },$$

where $L$ is the lower specification limit, $\mu$ is the process mean and $\sigma$ is the process standard deviation, which given by

4 $$\mu =\alpha {\gamma }^{\beta +1}B\left(\frac{1}{\beta }+1,\frac{\alpha {\gamma }^{\beta }}{2}+1\right){ }_{2}{F}_{1}\left(\frac{\alpha {\gamma }^{\beta }}{2}+1,\frac{1}{\beta }+1,\frac{1}{\beta }+\frac{\alpha {\gamma }^{\beta }}{2}+1,-1\right),$$

$${\sigma }^{2}=\alpha {\gamma }^{\beta +2}B\left(\frac{2}{\beta }+1,\frac{\alpha {\gamma }^{\beta }}{2}+1\right){ }_{2}{F}_{1}\left(\frac{\alpha {\gamma }^{\beta }}{2}+1,\frac{2}{\beta }+1,\frac{2}{\beta }+\frac{\alpha {\gamma }^{\beta }}{2}+1,-1\right)$$

5 $$- {\left(\alpha {\gamma }^{\beta +1}B\left(\frac{1}{\beta }+1,\frac{\alpha {\gamma }^{\beta }}{2}+1\right){ }_{2}{F}_{1}\left(\frac{\alpha {\gamma }^{\beta }}{2}+1,\frac{1}{\beta }+1,\frac{1}{\beta }+\frac{\alpha {\gamma }^{\beta }}{2}+1,-1\right)\right)}^{2}$$

where $B\left(n,m\right)$ and ${2F}_{1} (n,m;z ;x)$ denote the beta function and the hypergeometric function, respectively. To assess the lifetime performance of products,${C}_{L}$ for Omega distribution, we can use Eqs. (4) and (5) as,

6 $${C}_{L}=\frac{\alpha {k\gamma }^{\beta +1}-L}{\sqrt{\alpha {\gamma }^{\beta +2}B\left(\frac{2}{\beta }+1,\frac{\alpha {\gamma }^{\beta }}{2}+1\right){ }_{2}{F}_{1}\left(\frac{\alpha {\gamma }^{\beta }}{2}+1,\frac{2}{\beta }+1,\frac{2}{\beta }+\frac{\alpha {\gamma }^{\beta }}{2}+1,-1\right)-{\alpha }^{2}{{k}^{2}\gamma }^{2\beta +2}}},$$

where

$$-\infty <{C}_{L}<\frac{\alpha {k\gamma }^{\beta +1}}{\sqrt{\alpha {\gamma }^{\beta +2}{\text{B}}\left(\frac{2}{\beta }+1,\frac{\alpha {\gamma }^{\beta }}{2}+1\right)2{\text{F}}1\left(\frac{\alpha {\gamma }^{\beta }}{2}+1,\frac{2}{\beta }+1,\frac{2}{\beta }+\frac{\alpha {\gamma }^{\beta }}{2}+1,-1\right)-{\alpha }^{2}{{k}^{2}\gamma }^{2\beta +2}}},$$

$$k=B\left(\frac{1}{\beta }+1,\frac{\alpha {\gamma }^{\beta }}{2}+1\right){ }_{2}{F}_{1}\left(\frac{\alpha {\gamma }^{\beta }}{2}+1,\frac{1}{\beta }+1,\frac{1}{\beta }+\frac{\alpha {\gamma }^{\beta }}{2}+1,-1\right).$$

The hazard function $h(x)$ of the Omega distribution is defined as

7 $$h\left(x\right)=\alpha \beta {x}^{\beta -1}\frac{{\gamma }^{2\beta }}{{\gamma }^{2\beta }-{x}^{2\beta }}.$$

**Figure 2:**
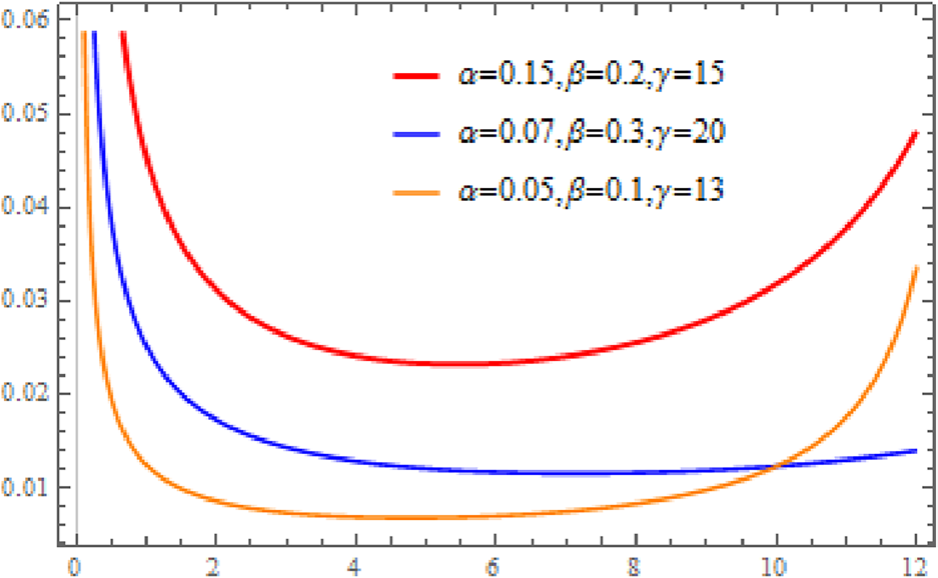
Bathtub-shaped omega hazard function plots.

Figure 2 shows plots of the Omega distribution hazard function. If $\beta <1$, then $h(x)$ is bathtub shaped.

## The conforming rate

When the lifetime of the product $X$ is greater than the lower specification limit $L$ , the product is referred to as a conforming product. The conforming rate is the realization of the ratio of the conforming product, which is described as

$${P}_{r}=p\left(X\ge L\right)=1-F(L)$$

8 $$={\left(\frac{{\gamma }^{\beta }+{\left(\alpha k{\gamma }^{\beta +1}-{C}_{L}{\left(\alpha {\gamma }^{\beta +2}B\left(\frac{2}{\beta }+1,\frac{\alpha {\gamma }^{\beta }}{2}+1\right){ }_{2}{F}_{1}\left(\frac{\alpha {\gamma }^{\beta }}{2}+1,\frac{2}{\beta }+1,\frac{2}{\beta }+\frac{\alpha {\gamma }^{\beta }}{2}+1,-1\right)-{\alpha }^{2}{k}^{2}{\gamma }^{2\beta +2}\right)}^{1/2}\right)}^{\beta }}{{\gamma }^{\beta }-{\left(\alpha {k}^{2}{\gamma }^{\beta +1}-{C}_{L}{\left(\alpha {\gamma }^{\beta +2}B\left(\frac{2}{\beta }+1,\frac{\alpha {\gamma }^{\beta }}{2}+1\right){ }_{2}{F}_{1}\left(\frac{\alpha {\gamma }^{\beta }}{2}+1,\frac{2}{\beta }+1,\frac{2}{\beta }+\frac{\alpha {\gamma }^{\beta }}{2}+1,-1\right)-{\alpha }^{2}{{k}^{2}\gamma }^{2\beta +2}\right)}^{1/2}\right)}^{\beta }}\right)}^{\frac{-\alpha \beta }{2}}.$$

Table 1 show the ${C}_{L}$ values v.s. the conforming rate ${P}_{r}$ values with $\left(\alpha ,\beta ,\gamma \right)$=$(\mathrm{4.28,2.61,57.79}).$ Clearly, there is a relation between the ${C}_{L}$ and the ${P}_{r}$. If the lifetime performance index ${C}_{L}$ increases, the conforming rate ${P}_{r}$ increases for given $\alpha , \beta$ and $\gamma$. The lifetime performance index ${C}_{L}$ is given as $2.3$ then, ${P}_{r}$ is equal to $1$. On the other hand, Table $1$ support for evaluating the lifetime performance of products in real example of Sect. "Testing process for the lifetime performance index"$.$

**Table 1:** The lifetime performance index ${{\text{C}}}_{{\text{L}}}$ v.s. the conforming rate ${{\text{P}}}_{{\text{r}}}.$

${C}_{L}$	${P}_{r}$	${C}_{L}$	${P}_{r}$	${C}_{L}$	${P}_{r}$	${C}_{L}$	${P}_{r}$
− 11	3.3836 × ${10}^{-28}$	− 3	0.00254	0.3	0.59403	0.9	0.80306
− 10	3.8328 × ${10}^{-23}$	− 2	0.02968	0.4	0.63169	0.95	0.81786
− 9	9.5215 × ${10}^{-19}$	− 1	0.16438	0.5	0.66861	1	0.83213
− 8	6.2976 × ${10}^{-15}$	− 0.5	0.30204	0.6	0.70448	1.5	0.94226
− 7	1.1904 × ${10}^{-11}$	− 0.25	0.38726	0.7	0.73901	2	0.99227
− 6	6.9219 × ${10}^{-9}$	− 0.00	0.47964	0.75	0.75569	2.1	0.9985
− 5	1.3370 × ${10}^{-6}$	0.1	0.51769	0.8	0.77195	2.2	0.9997
− 4	$0.00093$	0.2	0.55592	0.85	0.78774	2.3	1

## Maximum likelihood estimator of lifetime performance index

Consider the progressive type II censored sample is denoted by ${X}_{1:m:n}, {X}_{2:m:n},\dots \dots .,{X}_{m:m:n}$ with survival products ${R}_{1}, {R}_{2},\dots \dots .,{R}_{m}$ ejected from the life test. The likelihood function of this sample is given as (Casella and Berger^26^)

9 $$L\left(\theta \right)=C\prod_{i=1}^{m} { f}_{X}\left({x}_{i:m;n}|\theta \right){\left[1-{F}_{X}\left({x}_{i:m;n}|\theta \right)\right]}^{{R}_{i}},$$

where $$C=n(n-{R}_{1}-1).....(n-{R}_{1}-{R}_{2}-......-{R}_{m-1}-m+1),$$

${f}_{X}\left({x}_{i:m;n}|\theta \right)$ and ${F}_{X}\left({x}_{i:m;n}|\theta \right)$ are the pdf and cdf of $X$ given in Eqs. (1) and (2).

The likelihood function of ${X}_{1:m:n}, {X}_{2:m:n},\dots \dots .,{X}_{m:m:n}$ is given as,

$$L\left(\theta \right)=C\prod_{i=1}^{m}\alpha \beta {{x}_{i:m;n}}^{\beta -1}\frac{{\gamma }^{2\beta }}{{\gamma }^{2\beta }-{{x}^{2\beta }}_{i:m:n}}{(\frac{{\gamma }^{\beta }+{{x}^{\beta }}_{i:m:n}}{{\gamma }^{\beta }-{{x}^{\beta }}_{i:m:n}})}^{\frac{-\alpha {\gamma }^{\beta }}{2}\left({R}_{i}+1\right)},$$

10 $$=C{\left(\alpha \beta \right)}^{m}\prod_{i=1}^{m}{{x}_{i:m;n}}^{\beta -1}\frac{{\gamma }^{2\beta }}{{\gamma }^{2\beta }-{{x}^{2\beta }}_{i:m:n}}{(\frac{{\gamma }^{\beta }+{{x}^{\beta }}_{i:m:n}}{{\gamma }^{\beta }-{{x}^{\beta }}_{i:m:n}})}^{\frac{-\alpha {\gamma }^{\beta }}{2}\left({R}_{i}+1\right)},$$

where $$C=n\left(n-{R}_{1}-1\right).....\left(n-{R}_{1}-{R}_{2}-......-{R}_{m-1}-m+1\right).$$

The form of the log-likelihood function of this sample can be presented as,

11 $$l\left(\theta \right)=m\,\, ln\alpha +m\,\, ln\beta +(\beta -1)\sum_{i=1}^{m}ln({x}_{i:m:n})+\sum_{i=1}^{m}ln\left(\frac{{\gamma }^{2\beta }}{{\gamma }^{2\beta }-{{x}^{2\beta }}_{i:m:n}}\right)-\frac{\alpha {\gamma }^{\beta }}{2}\sum_{i=1}^{m}{R}_{i}ln\left(\frac{{\gamma }^{\beta }+{{x}^{\beta }}_{i:m:n}}{{\gamma }^{\beta }-{{x}^{\beta }}_{i:m:n}}\right).$$

By taking the first derivative of the Eq. (11) with respect to parameters $\alpha , \beta$ and $\gamma$ and be equal them to zero. Then we have these equations,

12 $$\frac{\partial l}{\partial \alpha }=\frac{m}{\alpha }-\frac{1}{2}{\gamma }^{\beta }\sum_{i=1}^{m}{R}_{i}ln\left(\frac{{\gamma }^{\beta }+{{x}^{\beta }}_{i:m:n}}{{\gamma }^{\beta }-{{x}^{\beta }}_{i:m:n}}\right),$$

$$\frac{\partial l}{\partial \beta }=\frac{m}{\beta }+\sum_{i=1}^{m}\mathit{ln}{(x}_{i:m:n})-\frac{1}{2}{\gamma }^{\beta }\alpha\,\, \mathit{ln}\,\,\gamma \sum_{i=1}^{m}{R}_{i}ln\left(\frac{{\gamma }^{\beta }+{{x}^{\beta }}_{i:m:n}}{{\gamma }^{\beta }-{{x}^{\beta }}_{i:m:n}}\right)+2\sum_{i=1}^{m}(\mathit{ln}\gamma -\frac{({\gamma }^{2\beta }\mathit{ln}\gamma -{x}_{i:m:n}^{2\beta }ln\left({x}_{i:m:n}\right))}{{\gamma }^{2\beta }-{x}_{i:m:n}^{2\beta }}$$

13 $$-\frac{1}{2}{\alpha \gamma }^{\beta }\sum_{i=1}^{m}\frac{{R}_{i}\left({\gamma }^{\beta }-{x}_{i:m:n}^{\beta }\right)\left({\gamma }^{\beta }\mathit{ln}\gamma +{x}_{i:m;n}^{\beta }ln\left({x}_{i:m:n}\right)\right)-{R}_{i}{x}_{i:m;n}^{\beta }\left({\gamma }^{\beta }+{x}_{i:m:n}^{\beta }\right)\left({\gamma }^{\beta }\mathit{ln}\gamma -ln\left({x}_{i:m:n}\right)\right)}{\left({\gamma }^{2\beta }-{x}_{i:m:n}^{2\beta }\right)},$$

14 $$\frac{\partial l}{\partial \gamma }=-\frac{1}{2}{\alpha \beta \gamma }^{\beta -1}\sum_{i=1}^{m}{R}_{i}ln\left(\frac{{\gamma }^{\beta }+{x}_{i:m;n}^{\beta }}{{\gamma }^{\beta }-{x}_{i:m:n}^{\beta }}\right)+\alpha {\beta \gamma }^{2\beta -1}\sum_{i=1}^{m}\frac{{R}_{i}{x}_{i:m:n}^{\beta }}{{\gamma }^{2\beta }-{x}_{i:m:n}^{2\beta }}-2\beta {\gamma }^{-1}\sum_{i=1}^{m}\frac{{x}_{i:m:n}^{2\beta }}{{\gamma }^{2\beta }-{x}_{i:m:n}^{2\beta }}.$$

The invariance property of the MLE is used to obtain the MLE of ${C}_{L}$(Zehan^27^). The MLE of ${C}_{L}$ can be constructed as follows,

15 $$\widehat{{C}_{L}}=\frac{\widehat{\alpha }\widehat{k}{\widehat{\gamma }}^{\widehat{\beta }+1}-L}{\sqrt{\widehat{\alpha }{\widehat{\gamma }}^{\widehat{\beta }+2}{\text{B}}\left(\frac{2}{\widehat{\beta }}+1,\frac{\widehat{\alpha }{\widehat{\gamma }}^{\widehat{\beta }}}{2}+1\right){ }_{2}{F}_{1}\left(\frac{\widehat{\alpha }{\widehat{\gamma }}^{\widehat{\beta }}}{2}+1,\frac{2}{\widehat{\beta }}+1,\frac{2}{\widehat{\beta }}+\frac{\widehat{\alpha }{\widehat{\gamma }}^{\widehat{\beta }}}{2}+1,-1\right)-{\widehat{\alpha }}^{2}{\widehat{k}}^{2}{\widehat{\gamma }}^{2\widehat{\beta }+2}}}.$$

Moreover, the asymptotic normal model for the MLEs is stated in the following technique (see Soliman^28^). From Eq. (12) we have

16 $$\frac{{\partial }^{2}l}{{\partial \alpha }^{2}}=-\frac{m}{{\alpha }^{2}},$$

17 $$\frac{{\partial }^{2}l}{\partial \alpha \partial \beta }=-\frac{1}{2}{\gamma }^{\beta }\mathit{ln}\gamma \sum_{i=1}^{m}{R}_{i}ln\left(\frac{{\gamma }^{\beta }+{{x}^{\beta }}_{i:m:n}}{{\gamma }^{\beta }-{{x}^{\beta }}_{i:m:n}}\right)-{\gamma }^{2\beta }\sum_{i=1}^{m}\frac{{R}_{i}{x}_{i:m:n}^{\beta }(ln\left({x}_{i:m:n}\right)-\mathit{ln}\gamma )}{{\gamma }^{2\beta }-{x}_{i:m:n}^{2\beta }},$$

18 $$\frac{{\partial }^{2}l}{\partial \alpha \partial \gamma }=-\frac{1}{2}\beta {\gamma }^{\beta -1}\sum_{i=1}^{m}{R}_{i}ln\left(\frac{{\gamma }^{\beta }+{{x}^{\beta }}_{i:m:n}}{{\gamma }^{\beta }-{{x}^{\beta }}_{i:m:n}}\right)+{\beta \gamma }^{2\beta -1}\sum_{i=1}^{m}\frac{{R}_{i}{x}_{i:m:n}^{\beta }}{{\gamma }^{2\beta }-{x}_{i:m:n}^{2\beta }}.$$

From Eqs. (13) and (14) we have,

$$\frac{{\partial }^{2}l}{{\partial \beta }^{2}}=-\frac{m}{{\beta }^{2}}-\alpha {\gamma }^{\beta }\mathit{ln}\gamma -\alpha {\gamma }^{\beta }\mathit{ln}\gamma \sum_{i=1}^{m}{R}_{i}ln\left(\frac{{\gamma }^{\beta }+{{x}^{\beta }}_{i:m:n}}{{\gamma }^{\beta }-{{x}^{\beta }}_{i:m:n}}\right)+\alpha {\gamma }^{\beta }\mathit{ln}\gamma \sum_{i=1}^{m}\frac{2{\gamma }^{\beta }\mathit{ln}\gamma -{R}_{i}{x}_{i:m:n}^{\beta }ln\left({x}_{i:m:n}\right)}{{\gamma }^{2\beta }-{x}_{i}^{2\beta }}$$

19 $$-\alpha {\gamma }^{\beta }\sum_{i=1}^{m}\frac{{\gamma }^{\beta }\mathit{ln}\gamma -{R}_{i}{x}_{i:m:n}^{\beta }ln\left({x}_{i:m:n}\right)({\gamma }^{2\beta }+{x}_{i:m:n}^{2\beta })}{{\left({\gamma }^{2\beta }-{x}_{i}^{2\beta }\right)}^{2}}+2\sum_{i=1}^{m}\frac{2{\gamma }^{2\beta }\mathit{ln}\gamma -{x}_{i:m:n}^{2\beta }ln\left({x}_{i:m:n}\right)}{{\left({\gamma }^{2\beta }-{x}_{i:m:n}^{2\beta }\right)}^{2}},$$

$$\frac{{\partial }^{2}l}{{\partial \gamma }^{2}}=-\frac{1}{2}\alpha \beta \left(\beta -1\right){\gamma }^{\beta -2}\sum_{i=1}^{m}{R}_{i}ln\left(\frac{{\gamma }^{\beta }+{{x}^{\beta }}_{i:m:n}}{{\gamma }^{\beta }-{{x}^{\beta }}_{i:m:n}}\right)+2\alpha {\beta }^{2}{\gamma }^{2(\beta -1)}\sum_{i=1}^{m}\frac{{R}_{i}{x}_{i:m:n}^{\beta }}{{\gamma }^{2\beta }-{x}_{i:m:n}^{2\beta }}$$

20 $$-\alpha {\beta \gamma }^{2\left(\beta -1\right)}\sum_{i=1}^{m}\frac{{R}_{i}{x}_{i:m:n}^{\beta }\left(\left(\beta +1\right){\gamma }^{2\beta }+\left(\beta -1\right){x}_{i:m:n}^{2\beta }\right)}{{\left({\gamma }^{2\beta }-{x}_{i:m:n}^{2\beta }\right)}^{2}}-2\beta {\gamma }^{-2}\sum_{i=1}^{m}\frac{{x}_{i:m:n}^{2\beta }\left({x}_{i:m:n}^{2\beta }-\left(2\beta +1\right){\gamma }^{2\beta }\right)}{{\left({\gamma }^{2\beta }-{x}_{i:m:n}^{2\beta }\right)}^{2}},$$

$$\frac{{\partial }^{2}l}{\partial \beta \partial \gamma }=-\frac{1}{2}\alpha {\gamma }^{\beta -1}\left(1+\beta \mathit{ln}\gamma \right)\sum_{i=1}^{m}{R}_{i}ln\left(\frac{{\gamma }^{\beta }+{{x}^{\beta }}_{i:m:n}}{{\gamma }^{\beta }-{{x}^{\beta }}_{i:m:n}}\right)+\alpha \beta {\gamma }^{2\beta -1}\mathit{ln}\gamma \sum_{i=1}^{m}\frac{{R}_{i}{x}_{i:m:n}^{\beta }}{{\gamma }^{2\beta }-{x}_{i:m:n}^{2\beta }}$$

$$+\alpha \beta {\gamma }^{2\beta -1}\sum_{i=1}^{m}\frac{{R}_{i}{x}_{i:m:n}^{\beta }(\mathit{ln}\gamma -ln\left({x}_{i:m:n}\right))}{{\gamma }^{2\beta }-{x}_{i:m:n}^{2\beta }}+2{\gamma }^{-1}\sum_{i=1}^{m}\frac{{x}_{i:m:n}^{2\beta }({\gamma }^{2\beta }(-1+2\beta \mathit{ln}\gamma -2\beta ln\left({x}_{i:m:n}\right))+{x}_{i:m:n}^{2\beta })}{{({\gamma }^{2\beta }-{x}_{i:m:n}^{2\beta })}^{2}}$$

21 $$-\alpha {\gamma }^{2\beta -1}\sum_{i=1}^{m}\frac{{R}_{i}{x}_{i:m:n}^{\beta }({\gamma }^{2\beta }(-1+\beta \mathit{ln}\gamma -\beta ln\left({x}_{i:m:n}\right))+(1+\beta \mathit{ln}\gamma -\beta ln\left({x}_{i:m:n}\right)){x}_{i:m:n}^{2\beta })}{{\left({\gamma }^{2\beta }-{x}_{i}^{2\beta }\right)}^{2}}.$$

The asymptotic normality results of the MLE of $\theta$ can be represented as,

22 $$\widehat{\theta }\sim N\left(\theta ,I{\left(\theta \right)}^{-1}\right),$$

where, $I\left(\theta \right)$ is the Fisher information matrix. The approximate information matrix ${I}_{0}\left(\widehat{x}\right)$ is given by,

23 $${I}_{0}\left(\widehat{\theta }\right)=\left[\begin{array}{ccc}\frac{{\partial }^{2}l}{{\partial \alpha }^{2}}& \frac{{\partial }^{2}l}{\partial \alpha \partial \beta }& \frac{{\partial }^{2}l}{\partial \alpha \partial \gamma }\\ \frac{{\partial }^{2}l}{\partial \beta \partial \alpha }& \frac{{\partial }^{2}l}{{\partial \beta }^{2}}& \frac{{\partial }^{2}l}{\partial \beta \partial \gamma }\\ \frac{{\partial }^{2}l}{\partial \gamma \partial \alpha }& \frac{{\partial }^{2}l}{\partial d\partial \beta }& \frac{{\partial }^{2}l}{{\partial \gamma }^{2}}\end{array}\right].$$

The variance–covariance matrix ${I}_{0}{(\widehat{\theta })}^{-1}$ is utilized to estimate ${I}_{0}{(\theta )}^{-1}.$ Assume that ${C}_{L}\equiv C(\theta )$, and the multivariate delta method identified that the asymptotic normal distribution of $C(\widehat{\theta })$ is

24 $$\widehat{{C}_{L}}\equiv C\left(\widehat{\theta }\right)\sim N\left({C}_{L}, {\psi }_{\theta }\right).$$

The approximate asymptotic variance–covariance matrix ${\psi }_{\widehat{\theta }}$ of $C\left(\theta \right)$ is utilized to estimate ${\psi }_{\theta }$ and it given by

25 $${\psi }_{\widehat{\theta }}=\left(\begin{array}{cc}\frac{\partial C\left(\theta \right)}{\partial \alpha }& \begin{array}{cc}\frac{\partial C\left(\theta \right)}{\partial \beta }& \frac{\partial C\left(\theta \right)}{\partial \gamma }\end{array}\end{array}\right) {I}_{0}{\left(\theta \right)}^{-1}\left(\begin{array}{c}\frac{\partial C\left(\theta \right)}{\partial \alpha }\\ \frac{\partial C\left(\theta \right)}{\partial \beta }\\ \frac{\partial C\left(\theta \right)}{\partial \gamma }\end{array}\right).$$

## Bayesian estimator of the lifetime performance index

Bayes estimation is a powerful and versatile technique in statistics that uses prior knowledge and observed data to estimate the value of unknown parameters. It’s based on Bayes' theorem, which provides a framework for updating beliefs based on new evidence. The gamma prior density function is shown as

26 $$\left\{\begin{array}{c}{h\left(\alpha \right)=\alpha }^{{b}_{1}-1}{e}^{-{c}_{1}\alpha },\alpha >0\\ h\left(\beta \right)={\beta }^{{b}_{2}-1}{e}^{-{c}_{2}\beta },\beta >0\\ h\left(\gamma \right)={\gamma }^{{b}_{3}-1}{e}^{-{c}_{3}d},\gamma >0\end{array}\right.$$

where ${b}_{i}$ and ${c}_{i} ,i=\mathrm{1,2},3$ are hyper-parameters which represents our initial beliefs about the possible values of the parameters.

By combining the prior distribution with the likelihood function using Bayes' theorem, you obtain the posterior distribution. The posterior is denoted by ${h}^{*}\left(\alpha ,\beta ,\gamma |x\right)$. When we combine Eqs. (10) and (26), we get posterior function as following,

27 $$h^{*} \left( {\alpha ,\beta ,\gamma |x} \right) = \frac{{h\left( \alpha \right)h\left( \beta \right)h(\gamma )L\left( {\alpha ,\beta ,\gamma |x} \right)}}{{\smallint _{0}^{\infty } \smallint _{0}^{\infty } \smallint _{0}^{\infty } h\left( \alpha \right)h\left( \beta \right)h(\gamma )L\left( {\alpha ,\beta ,\gamma |x} \right)d\alpha d\beta d\gamma }}.$$

Square error loss (SEL) function is crucial for guiding model training towards better performance. We used the SEL for $\theta =(\alpha ,\beta ,\gamma )$ which is given by,

28 $$L\left(\theta ,\widehat{\theta }\right)={(\theta -\widehat{\theta })}^{2}.$$

Hence, the Bayes estimate of a function of $\alpha ,\beta ,\gamma$, say $g\left(\alpha ,\beta ,\gamma \right)$ under the SEL function in Eq. (28).

29 $${\widehat{g}}_{BS}\left(\alpha ,\beta ,\gamma |x\right)={E}_{\alpha ,\beta ,\gamma |x}\left(g\left(\alpha ,\beta ,\gamma \right)\right) ,$$

And,

30 $$E_{{\alpha ,\beta ,\gamma |x}} \left( {g\left( {\alpha ,\beta ,\gamma } \right)} \right) = \frac{{\smallint _{0}^{\infty } \smallint _{0}^{\infty } \smallint _{0}^{\infty } g\left( {\alpha ,\beta ,\gamma } \right)h\left( \alpha \right)h\left( \beta \right)h(\gamma )L\left( {\alpha ,\beta ,\gamma |x} \right)d\alpha d\beta d\gamma }}{{\smallint _{0}^{\infty } \smallint _{0}^{\infty } \smallint _{0}^{\infty } h\left( \alpha \right)h\left( \beta \right)h(\gamma )L\left( {\alpha ,\beta ,\gamma |x} \right)d\alpha d\beta d\gamma }}.$$

In statistical analysis and prediction problems, a symmetric loss function, the linear exponential (LINEX) loss function is very helpful in many respects see Ref.^29^. It is derived as,

31 $$L\left(\Delta \right)=\left({e}^{\epsilon \Delta }-\epsilon \Delta -1\right) ,$$

where $\epsilon$ is a loss function scale parameter. LINEX loss function penalizing overestimation only linearly. This makes it suitable for situations where underestimation is considered more harmful than overestimation. The LINEX loss function can take positive or negative values for $\epsilon$ and it is close to zero.

The Bayesian estimate for $\alpha ,\beta ,\gamma$ is denoted by $\pi (\alpha ,\beta ,\gamma )$ under the LINEX function. $\pi (\alpha ,\beta ,\gamma )$ is derived as the following equation.

32 $${\widehat{\pi }}_{BS}\left(\alpha ,\beta ,\gamma |x\right)=\frac{-1}{\epsilon }{\text{log}}\left[E\left({e}^{-\epsilon \pi \left(\alpha ,\beta ,\gamma \right)}|x\right)\right],\epsilon \ne 0$$

And,

33 $$E\left({e}^{-\epsilon \pi \left(\alpha ,\beta ,\gamma \right)}|x\right)=\frac{{\int }_{0}^{\infty }{\int }_{0}^{\infty }{\int }_{0}^{\infty }{e}^{-\epsilon \pi \left(\alpha ,\beta ,\gamma \right)}h\left(\alpha \right)h\left(\beta \right)h(\gamma )L\left(\alpha ,\beta ,\gamma |x\right)d\alpha d\beta dd }{{\int }_{0}^{\infty }{\int }_{0}^{\infty }{\int }_{0}^{\infty }h\left(\alpha \right)h\left(\beta \right)h(\gamma )L\left(\alpha ,\beta ,\gamma |x\right)d\alpha d\beta d\gamma }.$$

Markov chain Monte Carlo (MCMC) is a powerful and versatile technique used in statistics and computational physics to sample from probability distributions. It is particularly useful for complex distributions where direct sampling is difficult or impossible. In this paper, the calculation of likelihood function is impossible. Hence, we must construct the joint posterior density function in order to the Bayesian estimation for ${C}_{L}$ by applying MCMC approach. The implementation of MCMC, a specific approach called Gibbs sampling within Metropolis is chosen. The joint posterior is computed from Eq. (27)

34 $${h}^{*}\left(\alpha ,\beta ,\gamma |x\right)\propto { \alpha }^{m+{b}_{1}-1}{ {\beta }^{m+{b}_{2}-1} {\gamma }^{{b}_{3}-1} e}^{-{c}_{1}\alpha -{c}_{2}\beta -{c}_{3}\gamma }\prod_{i=1}^{m}{{x}_{i:m;n}}^{\beta -1}\frac{{\gamma }^{2\beta }}{{\gamma }^{2\beta }-{{x}^{2\beta }}_{i:m:n}}{(\frac{{\gamma }^{\beta }+{{x}^{\beta }}_{i:m:n}}{{\gamma }^{\beta }-{{x}^{\beta }}_{i:m:n}})}^{\frac{-\alpha {\gamma }^{\beta }}{2}\left({R}_{i}+1\right)}$$

The conditional posterior densities of $\alpha ,\beta$ and $\gamma$ is calculated as follows:

35 $${{h}_{1}}^{*}\left(\alpha |\beta ,\gamma ,x\right)\propto {\alpha }^{m+{b}_{1}-1}{e}^{-{c}_{1}\alpha }\prod_{i=1}^{m}{\left(\frac{{\gamma }^{\beta }+{{x}^{\beta }}_{i:m:n}}{{\gamma }^{\beta }-{{x}^{\beta }}_{i:m:n}}\right)}^{\frac{-\alpha {\gamma }^{\beta }}{2}\left({R}_{i}+1\right)} .$$

36 $${{h}_{2}}^{*}\left(\beta |\alpha ,\gamma ,x\right)\propto {\beta }^{m+{b}_{2}-1}{e}^{-{c}_{2}\beta }\prod_{i=1}^{m}{{x}_{i:m;n}}^{\beta -1}\frac{{\gamma }^{2\beta }}{{\gamma }^{2\beta }-{{x}^{2\beta }}_{i:m:n}}{\left(\frac{{\gamma }^{\beta }+{{x}^{\beta }}_{i:m:n}}{{\gamma }^{\beta }-{{x}^{\beta }}_{i:m:n}}\right)}^{\frac{-\alpha {\gamma }^{\beta }}{2}\left({R}_{i}+1\right)}.$$

37 $${{h}_{3}}^{*}\left(\gamma |\alpha ,\beta ,x\right)\propto {\gamma }^{{b}_{3}-1}{e}^{-{c}_{3}\gamma } \prod_{i=1}^{m}\frac{{\gamma }^{2\beta }}{{\gamma }^{2\beta }-{{x}^{2\beta }}_{i:m:n}}{(\frac{{\gamma }^{\beta }+{{x}^{\beta }}_{i:m:n}}{{\gamma }^{\beta }-{{x}^{\beta }}_{i:m:n}})}^{\frac{-\alpha {\gamma }^{\beta }}{2}\left({R}_{i}+1\right)} .$$

The methodology of M-H is shown in the following steps,

- *Step 1*: Start with the first proposal ${(\alpha }^{(0)}, {\beta }^{\left(0\right)},{\gamma }^{(0)})$,
- *Step 2*: Assign j = 1.
- *Step 3*: Produce ${\alpha }^{(j)},{\beta }^{(j)}$ and ${\gamma }^{(j)}$ from ${{h}_{1}}^{*}\left({\alpha }^{j-1}|{\beta }^{j-1},{\gamma }^{j-1},x\right), {{h}_{2}}^{*}\left({\beta }^{j-1}|{\alpha }^{j-1},{\gamma }^{j-1},x\right)$ and ${{h}_{3}}^{*}\left({\gamma }^{j-1}|{\alpha }^{j-1},{\beta }^{j-1},x\right)$ from the M-H method with the normal distribution

$$N\left({\alpha }^{j-1}|var(\alpha )\right), N\left({\beta }^{j-1}|var(\beta )\right)$$ and $$N\left({\gamma }^{j-1}|var(\gamma )\right).$$

1. Derive ${\alpha }^{*}$ from $N\left({\alpha }^{j-1}|var(\alpha )\right), {\beta }^{*}$ from $N\left({\beta }^{j-1}|var(\beta )\right)$ and ${\gamma }^{*}$ from $N\left({\gamma }^{j-1}|var(\gamma )\right).$
2. Detect the probability of acceptance,
  $$\left\{\begin{array}{c}{\rho }_{\alpha }={\text{min}}\left[1,\frac{{{h}_{1}}^{*}\left({\alpha }^{*}|{\beta }^{\left(j-1\right)},{\gamma }^{\left(j-1\right)},x\right)}{{{h}_{1}}^{*}\left({\alpha }^{\left(j-1\right)}|{\beta }^{\left(j-1\right)},{\gamma }^{\left(j-1\right)},x\right)}\right]\\ {\rho }_{\beta }={\text{min}}\left[1,\frac{{{h}_{2}}^{*}\left({\beta }^{*}|{\alpha }^{\left(j-1\right)},{\gamma }^{\left(j-1\right)},x\right)}{{{h}_{2}}^{*}\left({\beta }^{\left(j-1\right)}|{\alpha }^{\left(j-1\right)},{\gamma }^{\left(j-1\right)},x\right)}\right]\\ {\rho }_{\gamma }={\text{min}}\left[1,\frac{{{h}_{3}}^{*}\left({\gamma }^{*}|{\alpha }^{\left(j-1\right)},{\beta }^{\left(j-1\right)},x\right)}{{{h}_{3}}^{*}\left({\gamma }^{\left(j-1\right)}|{\alpha }^{\left(j-1\right)},{\beta }^{\left(j-1\right)},x\right)}\right]\end{array}\right.$$
3. Generate ${u}_{1}, {u}_{2}$ and ${u}_{3}$ from a uniform $(\mathrm{0,1})$ distribution.
4. If ${u}_{1}<{\rho }_{\alpha },$ accept the suggestion and put ${\alpha }^{(j)}={\alpha }^{*}$ otherwise put ${\alpha }^{j}={\alpha }^{j-1}$.
5. If ${u}_{2}<{\rho }_{\beta },$ accept the suggestion and put ${\beta }^{(j)}={\beta }^{*}$ otherwise put ${\beta }^{j}={\beta }^{j-1}$.
6. If ${u}_{1}<{\rho }_{\gamma },$ accept the suggestion and put ${\gamma }^{(j)}={\gamma }^{*}$ otherwise.

Put $${\gamma }^{j}={\gamma }^{j-1}$$.

- Step 4: Count the ${{C}_{LBS}}^{(j)}as following$,
  38 $${{C}_{LBS}}^{(j)}=\frac{{\alpha }^{(j)}{{k}^{(j)}\gamma }^{(j)({\beta }^{(j)}+1)}-L}{\sqrt{{\alpha }^{(j)}{\gamma }^{(j)({\beta }^{(j)}+2)}B\left(\frac{2}{{\beta }^{(j)}}+1,\frac{{\alpha }^{(j)}{\gamma }^{(j)({\beta }^{(j)})}}{2}+1\right){ }_{2}{F}_{1}\left(\frac{{\alpha }^{(j)}{\gamma }^{{\beta }^{(j)}}}{2}+1,\frac{2}{{\beta }^{(j)}}+1,\frac{2}{{\beta }^{(j)}}+\frac{{\alpha }^{(j)}{\gamma }^{(j){\beta }^{(j)}}}{2}+1,-1\right)-{\alpha }^{2(j)}{{k}^{2(j)}\gamma }^{(j)(2{\beta }^{(j)}+2)}}}$$
- *Step 5*: Suppose that $j=j+1.$
- *Step 6*: Repeat N times steps from step 3 to step 5 to have ${\alpha }^{(i)},{\beta }^{(i)},{\gamma }^{(i)}$ and ${{{C}_{L}}^{(BS)}}^{(i)}, i=\mathrm{1,2},\dots ,N.$
- *Step 7*: Assess the credible interval of $\alpha ,\beta ,\gamma$ and ${C}_{L}$ order ${\alpha }^{(i)},{\beta }^{(i)},{\gamma }^{(i)}$ and ${{{C}_{L}}^{(BS)}}^{(i)}, i=\mathrm{1,2},..,N$ as ${\alpha }_{(1)}<{\alpha }_{(2)}<\dots <{\alpha }_{\left(N\right)}, {\beta }_{\left(1\right)}<{\beta }_{\left(2\right)}<\dots <{\beta }_{\left(N\right)}, {\gamma }_{(1)}<{\gamma }_{(2)}<\dots <{\gamma }_{\left(N\right)}$ and ${{C}_{L}}_{(1)}<{{C}_{L}}_{(2)}<\dots <{{C}_{L}}_{(N)}$ . Then, the $100(1-\rho )\%$ credible intervals of,

$$\vartheta =\left(\alpha ,\beta ,\gamma \right)$$ be $$\left(\varphi \left(N\left({\rho }_{\vartheta }/2\right)\right),\varphi \left(N\left(1-{\rho }_{\vartheta }/2\right)\right)\right)$$.

## The testing procedure for the lifetime performance index

The testing procedure allows us to measure the behavior of the lifetime of the product. In this section, a statistical testing process is submitted to evaluate whether the lifetime performance index achieves the pre-specified desired level ${c}^{*}$(target value). If the ${C}_{L}$ is larger than ${c}^{*}$, the product is categorized as reliable. The attitude of the lifetime test process is executed as follows.

The null hypothesis ${H}_{0}:{C}_{L} \le {c}^{*}$ is performed against an alternative hypothesis ${H}_{a}:{C}_{L}>{c}^{*} .$ The MLE for ${C}_{L}$ is applied as a test statistic. The rejection region can be represented as $\{\widehat{{C}_{L}} |\widehat{ {C}_{L}}>{C}_{0}\}$, when ${C}_{0}$ is a critical value. We can obtain the value of ${C}_{0}$ for assigned specified significance level $\alpha$ as follows,

39 $$P( \widehat{ {C}_{L}} > {C}_{0} | {C}_{L}= {c}^{*})=\alpha ,$$

40 $$P\left(\frac{\widehat{ {C}_{L}}-{C}_{L}}{\sqrt{{\psi }_{\widehat{\theta }}}}\le \frac{{C}_{0}-{c}^{*} }{\sqrt{{\psi }_{\widehat{\theta }}}}\right)=1-\alpha ,$$

where, $\frac{\widehat{{C}_{L}}-{C}_{L}}{\sqrt{{\psi }_{\widehat{\theta }}}}\sim N(\mathrm{0,1})$ and ${\psi }_{\widehat{\theta }}$ is the approximate asymptotic variance–covariance matrix given in Eq. (25)$.$ Then, the percentile of the standard normal distribution ${z}_{\alpha }=\frac{{C}_{0}-{c}^{*} }{\sqrt{{\psi }_{\widehat{\theta }}}}$ with right-tail probability $\alpha$ and the critical value can be performed as,

41 $${C}_{0}={c}^{*}+{z}_{\alpha }\sqrt{{\psi }_{\widehat{\theta }}.}$$

Moreover, the level $100\left(1-\alpha \right)\%$ one-sided confidence interval of ${C}_{L}$ is obtained as follows,

42 $${C}_{L}\ge \widehat{ {C}_{L}}-{z}_{\alpha }\sqrt{{\psi }_{\widehat{\theta }}.}$$

As a result, the $100\left(1-\alpha \right)\%$ ower confidence bound for ${C}_{L}$ can be performed as,

43 $$LB=\widehat{ {C}_{L}}-{z}_{\alpha }\sqrt{{\psi }_{\widehat{\theta }}}.$$

The methodology of the proposed testing procedure about ${C}_{L}$ can be employed in the next steps.

- *Step* $1$: Calculate the estimation of three parameters $\alpha , \beta$ and $\gamma$ of the Omega distribution. From Eqs. (12), (13) and (14) we can determine the MLE under progressive type-II censored sample ${X}_{1:m:n}, {X}_{2:m:n},\dots \dots .,{X}_{m:m:n}$ which is $\widehat{\alpha }=4.28$,$\widehat{\beta }=2.61$ and $\widehat{\gamma }=57.79.$
- *Step 2*: Detect the lower lifetime limit $L$ for the product and the target value ${c}^{*}.$
- *Step *$3$*:* Applying ${H}_{0}:{C}_{L} \le {c}^{*}$ which called the null hypothesis and the alternative hypothesis ${H}_{a}:{C}_{L}>{c}^{*}.$
- *Step 4* : Detect a significance level $\alpha .$
- *Step 5* : Conclude the $100\left(1-\alpha \right)\%$ one-sided confidence interval $[LB,\infty )$ for ${C}_{L}$ as,
  44 $$LB=\widehat{ {C}_{L}}-{z}_{\alpha }\sqrt{{\psi }_{\widehat{\theta }}},$$

where the number of observed failures before termination $m$ is determined also, the lower lifetime limit $L,$ the censoring scheme $R=\left({R}_{1}, {R}_{2},\dots \dots .,{R}_{m}\right)$ and the significance level.

- *Step 6*: The conclusion is detected as ${c}^{*}\notin [LB,\infty )$ then we will reject ${H}_{0}$. This indicates that the desired level for the performance of the product is reached.

Clearly, the hypothesis test process not only can assess the performance of products lifetime but also detect the customers' demands. In the following section two numerical examples obvious this concept.

## Simulation study

This section shows our simulation study involves generating data under various sample sizes.

- Number of samples: N = 1000.
- Sample sizes: n = 30, 50, 70, 90, 100, 200.

The equation $F\left(x\right)-u=0$, where $u$ is an observation from the Omega distribution and $F(x)$ is a cumulative distribution function of Omega distribution, is used to create this study. The following measures are assessed:

- Average bias of $\widehat{\alpha }, \widehat{\beta }$ and $\widehat{\gamma }$ of the parameters $\alpha , \beta$ and $\gamma$ are respectively:
  $$\frac{1}{N}\sum_{i=1}^{N}\left(\widehat{\alpha }-\alpha \right), \frac{1}{N}\sum_{i=1}^{N}\left(\widehat{\beta }-\beta \right) \,\,and\,\, \frac{1}{N}\sum_{i=1}^{N}\left(\widehat{\gamma }-\gamma \right) .$$
- The mean square error (MSE) of $\widehat{\alpha }, \widehat{\beta }$ and $\widehat{\gamma }$ of the parameters $\alpha , \beta$ and $\gamma$ are respectively:
  $$\frac{1}{N}\sum_{i=1}^{N}{\left(\widehat{\alpha }-\alpha \right)}^{2}, \frac{1}{N}\sum_{i=1}^{N}{\left(\widehat{\beta }-\beta \right)}^{2} \,\,and\,\, \frac{1}{N}\sum_{i=1}^{N}{\left(\widehat{\gamma }-\gamma \right)}^{2}.$$

From Table 2, we can know that the estimates have small bias and that the MSE decreases as the sample size increases. This suggests that the estimator is relatively consistent and that increasing the sample size can improve the accuracy of the estimates.

**Table 2:** Bias and MSE for parameters $\alpha , \,\,\beta\,\, and\,\, \gamma .$

$\alpha$	$\beta$	$\gamma$	N	Bias ($\alpha )$	MSE (α)	Bias ($\beta )$	MSE ($\beta )$	Bias ($\gamma )$	MSE ($\gamma )$
0.2	0.6	50	30	− 0.02079	0.001286	− 0.04186	0.003921	− 0.40384	0.305149
			50	− 0.01293	0.000757	− 0.02657	0.002246	− 0.37943	0.246498
			70	− 0.00803	0.000517	− 0.01869	0.001484	− 0.35149	0.202034
			90	− 0.00690	0.000430	− 0.01392	0.001188	− 0.32567	0.177657
			100	− 0.00647	0.000363	− 0.01268	0.001034	− 0.32148	0.175021
			200	− 0.00238	0.000185	− 0.00580	0.000479	− 0.25142	0.102086

## Applications

In this section we construct the testing procedure for the lifetime performance index on two applications. These two applications obviously show the importance of the Omega distribution on detecting the quality of products. One of them is about HPLC data and the other is about Ball Bearing data.

### HPLC data

High-performance liquid chromatography (HPLC) is an important mechanism for separating, identifying, and quantifying each component in the blood. Under large samples, Omega distribution fit to data set which is taken over 56 blood samples from an organ transplant recipient and analyzing an aliquot of each sample by a standard approved method high performance liquid chromatography (HPIC) (see Dube et. al.^30^).

A progressive type II censoring scheme was performed with $$m=24$$ and $${R}_{i}=\left({R}_{1}, {R}_{2},\dots \dots .,{R}_{m}\right) =(\mathrm{7,4}*\mathrm{2,0}* \mathrm{2,3},0*\mathrm{7,1},\mathrm{5,2},0*\mathrm{2,3}*\mathrm{2,0}*4).$$ Table 3 clarify the HPLC data.

**Table 3:** HPLC data.

99	327	203	241	578	153	109	156	93	244	245	71	151	271
125	275	350	521	370	166	402	35	77	428	185	112	206	130
127	285	221	336	339	129	318	159	104	198	254	280	266	653
109	298	440	340	162	227	556	118	159	980	346	118	148	87

We fitted the omega model to the data by employing maximum likelihood method. For determination stability with fitting of the model, we divided each observation by 980.

The Omega distribution is a relatively new lifetime distribution that has been shown to be a good fit for a variety of data sets, including HPLC data. The Omega distribution was compared to four other lifetime distributions, namely the Exponential^31^, Lindley^32^, gamma^33^, and TLAPE^34^ distributions, for their ability to fit HPLC data. K-S values and its *p*-values are shown in Table 4. The results of the study showed that the Omega distribution provided a better fit to the HPLC data than the other four distributions.

**Table 4:** Fitting results and various measures for several distributions to HPLC data.

Distribution	MLEs	KS	P-value
Exponential	$\widehat{\theta }=255.0$	0.2355	0.0040
Lindely	$\widehat{\theta }=0.0078$	0.0979	0.6567
Gamma	$\widehat{\alpha }=2.6882$	0.1007	0.6212
	$$\widehat{\beta }=94.858$$		
TLAPE	$\widehat{\alpha }=0.00375$	0.0970	0.6669
	$$\widehat{\beta }=3.26242$$		
Omega	$\widehat{\alpha }=7.39003$	0.0919683	0.730733
	$$\widehat{\beta }=1.6281$$		
	$$\widehat{\gamma }=1136.19$$		

Then, the life test procedure is constructed for ${C}_{L}$ of Omega distribution in the next steps.

- *Step 1*: Compute the MLE for parameters $\alpha , \beta$ and $\gamma$ of the Omega model under progressive type-II censoring sample. Table 5 shows censoring scheme under progressive type-II scheme.
- *Step 2*: Detect the lower limit specification L = 0.0191. The conforming rate of products should exceed 0.80306. The target value ${c}^{*}$ is equal to 0.9.
- *Step 3*: Constructing ${H}_{0}:{C}_{L} \le 0.9$ (null hypothesis) and the ${H}_{a}:{C}_{L}>0.9$ (alternative hypothesis).
- *Step 4*: Specify $\alpha =0.05$ significance level for calculating ${C}_{L}.$
- *Step 5*: Employing Eq. (43), the lower confidence interval bound.
  $$LB=2.64-1.645\sqrt{0.1115}=2.09237$$

**Table 5:** Censoring scheme for HPLC data.

$i$	1	2	3	4	5	6	7	8
${x}_{i:m:n}$	0.036	0.111	0.129	0.156	0.159	0.162	0.189	0.202
${R}_{i}$	7	4	4	0	0	3	0	0
$i$	9	10	11	12	13	14	15	16
${x}_{i:m:n}$	0.207	0.210	0.226	0.232	0.246	0.249	0.259	0.324
${R}_{i}$	0	0	0	0	0	1	5	2
$i$	17	18	19	20	21	22	23	24
${x}_{i:m:n}$	0.346	0.347	0.353	0.437	0.567	0.589	0.666	1
${R}_{i}$	0	0	3	3	0	0	0	0

So that, the 95% one-sided confidence interval for ${C}_{L}$ is $[LB,\infty )=[2.09237,\infty ).$

- Step 6: The performance index ${c}^{*}=0.9\notin [LB,\infty )=[2.09237,\infty )$ so we make a decition that null hypothesis ${H}_{0}:{C}_{L} \le 0.9$ is rejected. Moreover, $\widehat{{C}_{L}}=2.64>{c}^{*}+{z}_{\alpha }\sqrt{{\psi }_{\widehat{\theta }}}=0.9+1.645 \sqrt{0.1115 }\approx 1.44943$ So that, we accept the ${H}_{a}$ and the ${C}_{L}$ of the item adhere the desired level.

In Tables 6 and 7, it is seen that Bayes estimates outperform MLEs in the progressive type II samples. In comparison to the approximation confidence intervals, the Bayes credible intervals have the shortest confidence lengths. This study may effectively assist in the failure analysis of the HPLC dataset since the distribution of Omega distribution is well suited to the applicable data.

**Table 6:** Point estimates for the parameter $\alpha , \beta , \gamma$ and ${C}_{L}$ for HPLC data.

Parameter	MLE	SEL	LINEX		
			${c}_{1}=-2$	${c}_{2}=2$	${c}_{3}=0.001$
$\mathrm{\alpha }$	5.5639	5.8764	6.9842	6.2105	5.6782
$\upbeta$	1.4202	1.5524	1.9654	1.8345	1.4556
$\gamma$	1643.66	1835.72	2042.1	1852.32	1865.3
${C}_{L}$	1.3017	1.8976	2.3451	1.7357	1.8991

**Table 7:** The 95% asymptotic and credible intervals $\alpha , \beta , \gamma$ and ${C}_{L}$ for HPLC data.

Parameter	MLE	MCMC
$\mathrm{\alpha }$	(− 8.7679, 23.3679)	(0.1284, 20.3642)
$\upbeta$	(0.618937, 2.58106)	(0.7421, 2.3241)
$\gamma$	(− 1643, 18,739)	(0, 19,451)
${C}_{L}$	(0.7645, 2.1302)	(0.5541, 2.543)

### Ball bearing data

In a life test, ball bearing data devoted the number of millions of revolutions before failing for 23 ball bearings (see Lawless^35^). A progressive type II censoring scheme was presented with $m=11$ and ${R}_{i}=({R}_{1},{R}_{2},...,{R}_{m})=(0*\mathrm{3,3},\mathrm{2,3},\mathrm{1,0}*\mathrm{3,3}).$ Table 8, show the data on the failure times of 23 ball bearing.

**Table 8:** Failure times for 23 ball bearing data set.

0.1788	0.2892	0.3300	0.4152	0.4212	0.4560	0.4848	0.5184
0.5196	0.5412	0.5556	0.6780	0.6780	0.6780	0.6864	0.6864
0.6888	0.8412	0.9312	0.9864	1.0512	1.0584	1.2792	

We fitted the omega model to the data by employing maximum likelihood method. For determination stability with fitting of the model, we divided each observation by 1.2792.

Table 9 contains critical information for a comprehensive discussion around Ball Bearing data. Statistical tests like Kolmogorov–Smirnov test are applied to show which distribution is more fit to ball bearing data.Ahmadi et al.^36^ constructed the lifetime performance index with Weibull distribution. The Omega distribution performed better than the Weibull distribution because the Weibull distribution is not fitted to ball bearing data.

**Table 9:** Fitting results and various measures for several distributions to ball bearing data.

Distribution	MLEs	KS	P-value
Weibull	$\widehat{\alpha }=0.52225$	0.741731	$0.00204214$
	$$\widehat{\beta }=14.6739$$		
Kumerswamy	$\widehat{\alpha }=2.49066$	0.175724	0.476412
	$\widehat{\beta }=1$ 1.3687		
Power lomax	$\widehat{\alpha }=1.2632$	0.169049	0.52679
	$$\widehat{\beta }=2.5295$$		
	$$\widehat{\gamma }=1.00939$$		
Omega	$\widehat{a}=13.8007$	0.166612	0.545698
	$$\widehat{\beta }=2.61496$$		
	$$\widehat{\gamma }=50.3162$$		

Also, we can notice that the Omega distribution provided better than some other distribution on evaluating performance for products such as Kumaraswamy distribution^37^ and Power Lomax distribution^38^.

Then, we create life test procedure of ${C}_{L}$ for Omega distribution in the next steps.

**Table 10:** Type-II progressive censored sample failure times of the ball bearing data.

$i$	1	2	3	4
${x}_{i:m:n}$	0.139775	0.226079	0.257974	0.324578
${R}_{i}$	3	0	0	3
$i$	5	6	7	8
${x}_{i:m:n}$	0.405253	0.434334	0.536585	0.538462
${R}_{i}$	2	3	1	0
$i$	9	10	11	
${x}_{i:m:n}$	0.657598	0.727955	0.771107	
${R}_{i}$	0	0	3

- *Step 1*: Calculate the MLE for parameters $\alpha , \beta$ and $\gamma$ of the Omega model under progressive type-II censoring sample. Table 10 illustrates the progressive type-II censored scheme.
- *Step 2*: Determine the lower limit specification ${\text{L}}= 0.0732$. The conforming rate of products should be exceeding $0.80306$. The target value ${c}^{*}$ is equal to $0.9$.
- *Step 3*: Applying ${H}_{0}:{C}_{L} \le 0.9$ (null hypothesis) and the ${H}_{a}:{C}_{L}>0.9$ (alternative hypothesis).
- *Step 4*: Specify $\alpha =0.05$ significance level for calculating ${C}_{L}.$
- *Step 5*: utilizing Eq. (43), the lower confidence interval bound.
  $$LB=5.5-1.645\sqrt{1.50451}=3.48227$$

So that, the 95% one-sided confidence interval for ${C}_{L}$ is $[LB,\infty )=[3.48227,\infty ).$

- *Step 6*: Since, the performance index ${c}^{*}=0.9\notin [LB,\infty )=[3.48227,\infty )$ so we decide that null hypothesis ${H}_{0}:{C}_{L} \le 0.9$ is rejected. Moreover, $\widehat{{C}_{L}}=5.5>{c}^{*}+{z}_{\alpha }\sqrt{{\psi }_{\widehat{\theta }}}=0.9+1.645 \sqrt{1.50451 }\approx 2.91773$ So that, we accept the ${H}_{a}$ and the ${C}_{L}$ of the product adhere the desired level.

In Tables 11 and 12, it is seen that Bayes estimates outperform MLEs in the progressive type II samples. In comparison to the approximation confidence intervals, the Bayes credible intervals have the shortest confidence lengths. This study may effectively assist in the failure analysis of the Ball Bearing dataset since the distribution of Omega distribution is well suited to the applicable data.

**Table 11:** Point estimates for the parameter $\alpha , \beta , \gamma$ and ${C}_{L}$ for ball bearing data.

Parameter	MLE	SEL	LINEX		
			${c}_{1}=-2$	${c}_{2}=2$	${c}_{3}=0.001$
$\mathrm{\alpha }$	149.87	146.659	149.128	141.823	146.659
$\upbeta$	3.7787	3.75396	3.75402	3.75401	3.7539
$\gamma$	9.3494	9.406	9.8004	9.5352	9.2060
${C}_{L}$	2.35305	2.8451	2.9982	2.9745	2.4354

**Table 12:** The 95% asymptotic and credible intervals $\alpha , \beta , \gamma$ and ${C}_{L}$ for ball bearing data.

Parameter	MLE	MCMC
$\mathrm{\alpha }$	(− 72.8793, 372.62)	(140.688, 150.219)
$\upbeta$	(2.5403, 5.0172)	(3.74071, 3.7754)
$\gamma$	(− 25.5335, 26.8353)	(22.486, 28.035)
${C}_{L}$	(− 2.6803, 7.3864)	(0.8452, 3.2821)

## Conclusion

Evaluating the lifetime performance index is a critical point in our life to meet customers' demands. Making that assessment under censoring data allows good results in detecting the required customer level of quality. Some statistical measures are constructed to calculate ${C}_{L}$. By using the MLE method of ${C}_{L}$, we can test the ${C}_{L}$ of Omega distribution based on Progressive type-II censoring sample data with displaying the multivariate delta method. Bayes estimation based the Markov chain Monte Carlo (MCMC) method is performed for unknown parameters and ${C}_{L}$. To compare MCMC with MLE, a simulation study is provided. The simulation study is provided to compare MCMC with MLE. We compare Omega distribution with some other distributions by applying Kolmogorov–Smirnov test to show the superiority of Omega distribution. This analysis is derived for various values of $\alpha , \beta$ and $\gamma .$ Finally, the theoretical results are applied to two real data sets HPLC data and Ball Bearing data for specifying the desired concept of this paper.

In future research, as an alternative, Expectation–Maximization (EM) algorithm will be applied to estimate the parameters of Omega distribution. Whereas it's applied in various statistical areas, including mixture models, hidden Markov models, factor analysis, and many more. The EM algorithm is an iterative algorithm for estimating parameters. It consists of iterating the expectation step (E), which computes a bound for the loglikelihood function using the current parameter values, and the maximization step (M), where the bound is maximized with respect to parameters, we will discuss it in detail in our future research.

## Data Availability

The data that support the findings of this study are available within the article.
